# Comparison of efficacy of intravenous labetalol and intravenous hydralazine for management of pre-eclampsia in pregnant women

**DOI:** 10.4314/ahs.v23i1.34

**Published:** 2023-03

**Authors:** Mauzma Kausar, Samia Husain, Rubina Hussain

**Affiliations:** 1 Department of Obstetrics and Gynaecology, United Medical College Hospital, Karachi, Pakistan; 2 Department of Obstetrics and Gynaecology, Aziz medical center, Karachi, Pakistan; 3 Department of Obstetrics and Gynaecology, Ziauddin University Hospital, Karachi, Pakistan

**Keywords:** Preeclampsia, bolus dose, Hydralazine, Labetalol

## Abstract

**Objectives:**

To compare the efficacy of intravenous Labetalol and intravenous Hydralazine in reduction of blood pressure in patients with severe pre-eclampsia.

**Methodology:**

This comparative study was conducted at the Department of Obstetrics and Gynecology at Ziauddin University Hospital, Karachi from1st June 2019 to 30th June 2020. Total 208 pregnant women having severe pre-eclampsia (systolic pressure ≥160 mmHg and diastolic pressure ≥110mmHg) were included in study. Group A received I/V Labetalol. Group B received I/V Hydralazine. Efficacy of drugs was observed by reduction in blood pressure and the number of doses administered. Data was analysed using SPSS version 26.

**Results:**

Systolic blood pressure reduction in Labetalol group was significantly lower than in hydralazine group (105.5 ±11.3 vs. 115.8 ±17.1, p≤ 0.001). Diastolic blood pressure reduction was also lower in labetalol group than in hydralazine group (p= 0.03). Number of dosage of drugs in Group A (Labetalol) was 3.2 ±1.2 vs. Group B (Hydralazine) was 4.4±1.4, p =0.006).

**Conclusion:**

The results of this study show that Labetalol is more effective as compared to Hydralazine in terms of reducing the systolic and diastolic blood pressure and number of doses (Drugs) for in patients with severe preeclampsia.

## Introduction

Pre-eclampsia is a disorder occurring during pregnancy mostly in second trimester and if not treated, it develops to eclampsia.^1^ Pre-eclampsia in pregnant females is one of the leading causes of maternal, fetal and neonatal morbidity and mortality globally.^2^–^4^ In developed countries the frequency of pre-eclampsia is 3-8%^5^, ^6^ and in developing countries ranges from 2-17%.(1) Pre-eclampsia can be classified into gestational hypertension, pre-eclampsia, chronic hypertension, preeclampsia superimposed on chronic hypertension.^7^ Pre-eclampsia complicates 3-5% of all pregnancies and causes placental and vascular dysfunction that results in adverse outcomes as severe hypertension, stroke, seizure(eclampsia), renal and hepatic injury, intrauterine fetal growth restriction, even death.^8^

Progression from mild to severe hypertension in pregnancy is not predictable and sometimes can be sudden. Pregnancies with severe hypertension with systolic blood pressure >160mmHg and diastolic blood pressure >110mmHg needs admission in hospital.^9^ Treatment options for severe hypertension of pregnancy are Labetalol (oral or intravenous), intravenous hydralazine hydrochloride or oral nifedipine as first line anti-hypertensive therapy in critical care setting.^10^ Hydralazine hydrochloride is an arterial vasodilator and takes 20-30 min to be effective, reaches its peak level of effectiveness in 30-60 minutes. Labetalol onset timing is 5-10 minutes, reaches its peak level in 5-15minutes.^11^ When intravenous hydralazine hydrochloride was compared with other antihypertensive drugs like intravenous (I/V) labetalol, nifedipine, intravenous hydralazine was having more side effects such as maternal hypotension, Caesarean section rate and fetal heart rate abnormalities. When compared with intravenous labetalol specifically in terms of acute control of blood pressure, trial found no difference in persistent severe hypertension (5% in each group) but maternal hypotension was observed more in hydralazine hydrochloride treated women (2/100) as compared to (0/100) women treated with labetalol.^12^ Labetalol (non-selective alpha and beta blocker) gained more popularity as there is lower incidence of maternal hypotension and other side effects therefore it is preferable to hydralazine.^13^

Due to limited data from the local population, the debate is still ongoing in our setting. Hence, the aim of the current study was to compare the efficacy of Labetalol and Hydralazine for the treatment of hypertension in patients with preeclampsia. As most of the previous studies reported mean reduction in blood pressure in severe pre-eclampsia, we wanted to confirm if this is true for our population. Only a few studies focused on assessing the efficacy. This study will provide current & local magnitude of efficacy in comparison of labetalol and hydralazine in addition this study will emphasize on early diagnosis in making strategies to overcome the pregnancy related complications & morbidity.

## Material and Method

This comparative study was conducted at the Department of Obstetrics and Gynecology at Ziauddin University Hospital, Karachi during the period of 1^st^ June 2019 till 30^th^, June 2020. Total 208 pregnant women with 20-35 years of age, gestational age ≥20weeks (determined by dating scan (9-13weeks) having severe pre-eclampsia (systolic pressure ≥160 mmHg and diastolic pressure ≥110mmHg), admitted and fulfilling the criteria were included in study. Hypertension prior to pregnancy, women with renal disease, known allergy to hydralazine/labetalol, deranged LFT'S due to any other cause, were excluded from the study.

By using WHO Sample size calculator, taking efficacy of labetalol 86.5% 15 and hydralazine 96.2%. 15 Power of test 80%, level of significance 5%, then the estimated sample size was at least n=103 in each group and total sample size were at least n=206.

Two groups A and B of pregnant women were identified. The whole procedure was explained about the drugs used and procedure before taking Informed consent from the patient or her attendants. The study was approved by the ethical review committee of the institution.

a). Group A was received I/V Labetalol (112 participants)

b). Group B was received I/V Hydralazine (106 participants)

Nonprobability sampling technique was used for sample collection. Eligible women were given verbal explanations and written informed consent forms about the study. Enrolled patient's blood pressure was recorded by using Mercury manometer. An appropriate cuff size was used for accuracy. Age, parity, and gestational age were matched to control confounding variables. Blood pressure, pulse, respiratory rate, temperature, fetal heart rate was noted. General physical (including pedal edema and reflexes) and Systemic examination were performed) to make accurate diagnosis. Protein in urine was checked by dipstick method.

The patients were assigned to either of the group A or B. Group A was given 20mg bolus dose of labetalol in 2 minutes, and blood pressure was checked after 20 minutes, if not effective, then 40mg dose of labetalol was given. If still no effect was seen, then 80 mg were given every 20 minutes up to a maximum dose of 300mg and every time blood pressure was checked after 20 minutes. Group B were given I/V hydralazine 5mg in blouse dose then were repeated every 20 minutes in needed up to five doses, maximum 25mg were given. Target Blood pressure was 130/90mmHg.Throughout this period vitals were taken. Fetal hearts were closely monitored. Time taken to reduce the blood pressure up to desired level was noted on predesigned proforma. Final outcomes were measured after six hours in terms of efficacy.

Information was entered in SPSS (statistical packages of social sciences) version 240. Mean +SD and confidence interval were calculated for age, gestational age, parity, blood pressure and number of doses. Frequency and percentage were calculated for efficacy. Chi-square test with 90% confidence intervals was applied to compare the efficacy in both groups. Relative risk was calculated to estimate the magnitude of efficacy that is i.e., efficacy= 1-RR. Confounders were controlled through a stratification of age, parity, gestational age and number of doses to see the effect of these on outcome variables. Post stratification, apply chi-square test, taking P <0.05 as significant.

## Results

In this study the mean age of women age was 26.6±6.0 (mean ±S. D) years. The age range was 18-45 years. In Group A (Labetalol), the mean age (S. D) was 27.2±6.1 years and the range were 18-45 years; In Group B (Hydralazine), the mean age was 27.1±5.9 year with the age range was 18-45 years. Forty four percent women were belonging to ≤ 25 years of age group, as depicted in Table I. The mean gestational amenorrhea was 37.4±2.3. Booked patients were 96 % and multigravida was 73%. Eighty four percent patients had no history of miscarriage. Labetalol was administered to 51% patients and 49% received hydralazine. The efficacy of drug A Labetalol was 54.5%. (Table I)

**Table 1:** Baseline Characteristics of Pre-eclamptic in pregnant women (n=218)

Characteristic	n (%)
**Demographic**	
**Maternal age (year)** Mean(S.D)	26.6±6.0
≤ 25	95(43.6)
26–30	66(30.3)
≥ 31	57(26.1)

**Hospital Booking Status**	
Booked	209(95.9)
Un-booked	9(4.1)

**Obstetric characteristics**	
Primigravida	59(27.1)
Multigravida	159(72.9)

**Miscarriages**	
No miscarriage	184(84.4)
≤1 miscarriage	34(15.6)
**Gestational Amenorrhea (week)** Mean (S.D)	37.4 ±2.3

**Intravenous Drugs Groups**	
Labetalol(A)	112(51.4)
Hydralazine(B)	106(48.6)

**Drug Efficacy**	
Labetalol (A)	110(54.5)
Hydralazine (B)	92(45.5)

Mean, Standard deviation of Systolic blood pressure reduction in Group A (Labetalol) was 105.5±11.3 vs. Group B (Hydralazine) was 115.8 ±17.1, value of ‘p’ being 0.000 which is significant. Diastolic blood pressure reduction Mean, Standard deviation in Group A (Labetalol) was 69.5 ±9.2 vs. Group B (Hydralazine) was 78.9 ±11.2, value of ‘p’ being 0.003 which is statistically significantly high. (Table II)

**Table II:** Mean differences in Number of doses, gestational amenorrhea, and systolic and diastolic Blood Pressure Reduction in both Pre-eclamptic in pregnant women group n =218 (Labetalol & Hydralazine)

Variables	Labetalol (A) N (Mean ± SD)	Hydralazine (B) N (Mean ± SD)	Mean Difference	P value
**Systolic Pressure**	112 (105.5 ± 11.3)	106 (115.8 ± 17.1)	10.31	0.000
**Diastolic Pressure**	112 (69.5 ± 9.2)	106 (78.9 ± 11.2)	9.35	0.003
**Number of Doses**	112(3.2 ± 1.2)	106 (4.4 ± 1.4)	1.1	0.006
**Gestational Amenorrhea**	112 (37.1 ± 2.7)	106 (37.7 ± 1.7)	0.70	0.000

Number of dosage of drugs in Group A (Labetalol) was 3.2 ±1.2 (Mean, S.D) vs. Group B (Hydralazine) was 4.4±1.4, the P value was more than 0.05 (P value 0.006) was significantly high. Gestational amenorrhea Mean ±Standard deviation in Group A (Labetalol) was 37.1±2.7 vs. Group B (Hydralazine) was 37.7±1.7, P value 0.000 was more than 0.05 which was significant. (Table II)

Labetalol found more effective (54.5) as compared to Hydralazine (45.5%) in terms of reducing the systolic and diastolic blood pressure and number of doses (Drugs) for in patients with severe preeclampsia. (Figure I)

**Figure I:**
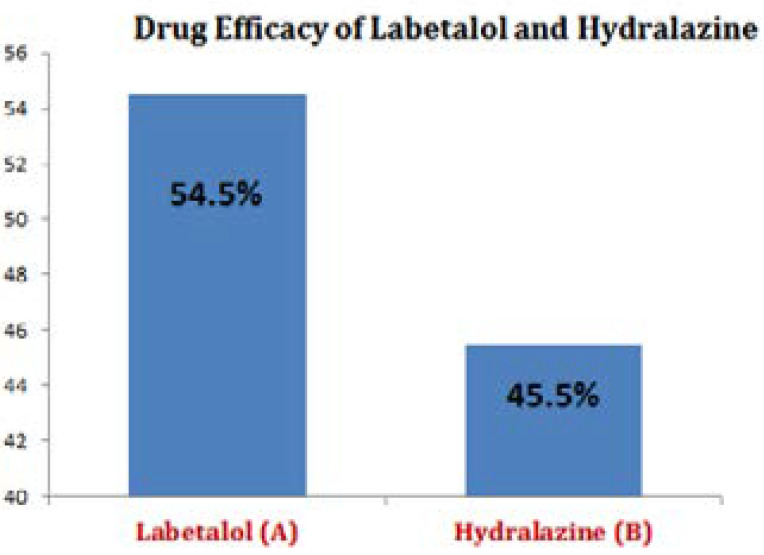
Drug efficacy of labetalol and hydralazine in pre-eclamptic pregnant women.

## Discussion

Pre-eclampsia is one of the leading causes of maternal mortality in the postpartum period. The current study evaluated the efficacy of hydralazine for the treatment of preeclampsia as compared to labetalol. We found that both drugs were effective as antihypertensive in patients with preeclampsia however, labetalol was more efficacious than hydralazine (p<0.000). Moreover, Hydralazine required significantly higher number of doses to control blood pressure compared to labetalol (P value 0.006). This indicates that despite the similar effectiveness of both the drugs, labetalol requires lesser dosage to treat hypertension in pregnant patients with preeclampsia hence, lesser probability of having side effects in the peripartum or postpartum period.

These findings are in accordance with previous literature. In a study by Nombur , it was found that both labetalol and hydralazine were equally effective for the treatment of severe hypertension in patients with preeclampsia.^4^ In another study, the safety and efficacy of intravenous labetalol and intravenous hydralazine were evaluated for the correction of hypertension during pregnancy.^14^ It was found that both labetalol and hydralazine were effective in lowering blood pressure in pregnant women. Most common complaints with hydralazine were palpitations and tachycardia in mothers. Neonatal hypotension and bradycardia were significantly more prominent in the labetalol group as compared to hydralazine group. In our study however, only mild headaches were experienced by patients. No other complications were reported by the patients.

In 2018, a network meta-analysis and trial sequential analysis was performed to analyse the current evidence on the most effective drugs used to treat hypertension during pregnancy.(15) A total of 51 studies were analysed for systematic review while 46 of these were eligible for meta-analysis. It was concluded that nifedipine, hydralazine and labetalol shared similar efficacy and safety profiles in the treatment of hypertension in pregnancy.

In contrast to the above-mentioned studies, a previous study evaluating the efficacy of hydralazine and labetalol for treatment of severe hypertension in pregnancy reported the efficacy of hydralazine & labetalol was 96.2% and 86.5% respectively.^2^ This could be because of the different routes of administration of drugs or the different number of doses administered to patients. The patients with multiple comorbidities may have different experiences with these drugs. Vigil de Gracia reported that both labetalol and hydralazine were effective in controlling the blood pressure in women in the postpartum period as well. 16

Both drugs are effective against severe hypertension in pregnancy however, in mild to moderate hypertension the doses should be carefully monitored to avoid maternal and neonatal complications. Another drug nifedipine has been used widely to treat hypertensive crisis in pregnant women with severe preeclampsia. Shi DD et al., observed that oral nifedipine was quicker in achieving blood pressure control in pregnant women as compared to intravenous labetalol. 17

Despite the many strong points of the study, there are some limitations. Firstly, the patients were not followed up to record long term side effects of the drugs. Secondly, it was a single-center study. A more diversified study could highlight some significant socio-demographic and ethnic factors at play. In the current study, no short-term complications were observed. However, further longitudinal studies should be conducted to evaluate the long-term side effects of these antihypertensive drugs on pregnant women with preeclampsia.

Pregnancy-induced hypertension and preeclampsia are lethal yet manageable complications of pregnancy and is a common occurrence in our setting. Adequate pharmacological intervention is necessary to lower the risk of complications in women with hypertensive diseases during pregnancy or delivery. According to our findings, labetalol, and hydralazine both give satisfactory results. However, based on lower requirement of dosage and higher efficacy rate, labetalol is considered more efficacious.

## Conclusion

The results of this study show that Labetalol is more effective as compared to Hydralazine in terms of reducing the systolic and diastolic blood pressure and number of doses (Drugs) for in patients with severe preeclampsia.
